# Remote sensing and process attribution uncertainties in the Dharali event

**DOI:** 10.1038/s44304-026-00211-w

**Published:** 2026-04-17

**Authors:** Anshuman Bhardwaj, Lydia Sam, Sheikh Nawaz Ali, Rayees Ahmed

**Affiliations:** 1 School of Geosciences, University of Aberdeen, Aberdeen, UK; 2 Birbal Sahni Institute of Palaeosciences, Lucknow, India; 3 Indian Institute of Science, Bengaluru, India

**Keywords:** Climate sciences, Natural hazards, Solid Earth sciences

## Abstract

A recent study by Dandabathula et al. attributes the 5 August 2025 Dharali disaster to an ice-patch collapse based largely on satellite imagery. Here, we examine remote sensing and process-attribution uncertainties in that interpretation. The proposed mechanism by Dandabathula et al. lacks spectral validation, geomorphic consistency, volumetric support, and geophysical corroboration. Available independent observations instead indicate rainfall-triggered mobilisation of unconsolidated paraglacial sediments, underscoring the need for rigorous process attribution in Himalayan hazard assessment.

The article by Dandabathula et al.^1^ attributes the Dharali disaster on 5 August 2025, in the Bhagirathi Basin of the India Himalaya, to an “ice-patch collapse” based primarily on interpretation of satellite imagery. However, several aspects of the remote sensing analysis and process attribution underlying this interpretation remain questionable. In particular, the proposed mechanism lacks robust validation of surface and glaciological conditions and is not supported by independent geomorphic or process-based evidence. The analysis^1^ relies heavily on a single, poorly constrained post-event optical image from 12 August 2025, that appears to lack a clear view of the hanging glacier, potentially giving the impression to Dandabathula et al.^1^ that the ice body had collapsed. It also does not sufficiently evaluate alternative mechanisms that are more consistent with collapse of rainfall-saturated paraglacial deposits, and the channel erosion and deposition patterns of several near-synchronous and proximal events along the Bhagirathi River, matching typical mudflow dynamics. As a result, the cryospheric trigger proposed for the Dharali event remains insufficiently supported and may lead to a mischaracterisation of the event’s origin, process chain, and associated hazard implications. Accurate process attribution is essential for guiding future research priorities and for developing reliable hazard assessment frameworks in rapidly changing mountain environments. Mischaracterising the underlying trigger risks shifting both scientific investigation and policy responses away from the processes that actually govern disaster risk in the Himalayan valleys.

## Mischaracterisation of the proposed source

**Fig. 1:**
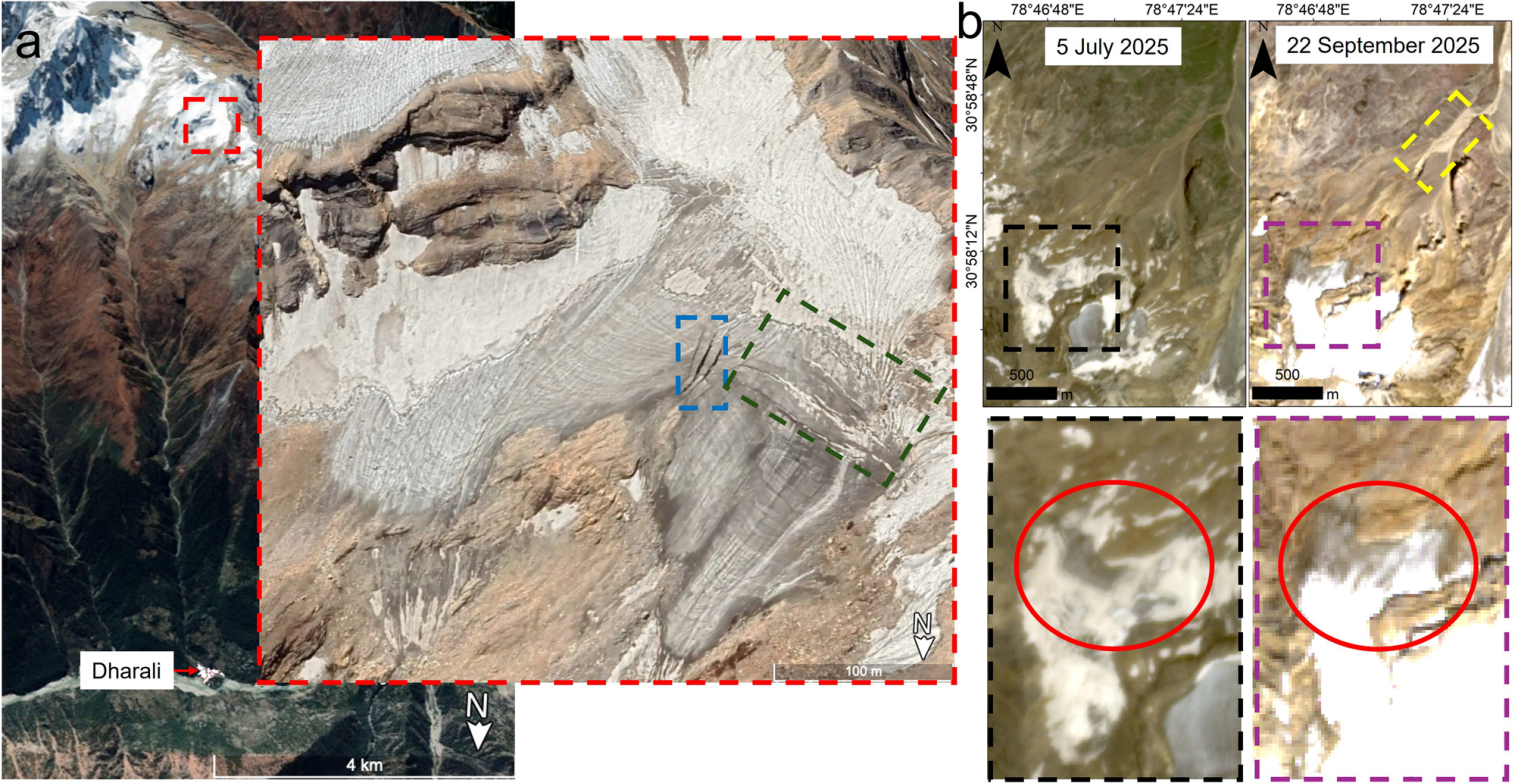
Surface characteristics in the upper catchment. **a** The blue rectangle indicates longitudinal crevasses, while the green rectangle highlights transverse crevasses. This 25 August 2014 high-resolution image is taken from Google Earth (CNES/Airbus). **b** The red ellipse marks the terminal portion of the glacier with exposed ice, which remains intact in both the pre- and post-event images. The pre-event image (3 m/pixel resolution) is from PlanetScope (Image ID: 20250705_055525_43_2538_3B_AnalyticMS_SR_8b) while the post-event image (10 m/pixel resolution) is from Sentinel 2 C (Image ID: S2C_MSIL2A_20250922T051701_N0511_R062_T44RKV). The yellow rectangle shows the collapsed paraglacial deposits in the post-event image.

What the authors^1^ describe as a “nivation zone with exposed ice patches” (Fig. 1a in Dandabathula et al.^1^) is, in fact, a hanging glacier. The high-resolution imagery presented in our Fig. 1a clearly shows fractures within this ice body that are diagnostic glacier crevasses, unequivocally demonstrating that this is an actively deforming mass of ice rather than an assemblage of stagnant or dead ice patches. Both longitudinal and transverse crevasses are visible, indicating a complex, high-stress regime in which the glacier is undergoing simultaneous longitudinal extension (acceleration) and lateral spreading^2^. An ice patch is a stationary, shallow accumulation of snow and ice that persists year-round but does not move^3^, whereas a hanging glacier is a mass of ice, often remnant from a larger glacier, that hangs on steep mountain slopes and moves, frequently calving ice into the valley below^4^.

Furthermore, Fig. 1b shows intact glacier ice with a distinctly darker albedo in both pre- and post-event imagery. The only observable change between the two dates is the spatial extent of seasonal snow cover on and around the glacier. If, as Dandabathula et al.^1^ assert, the entire “ice patch” had collapsed and triggered the Dharali event, the persistence of exposed glacier ice in the 22 September 2025 image (Fig. 1b) would be physically inconsistent with such a failure scenario. The only significant and confirmed surface change close to this glacier is highlighted by the yellow rectangle in Fig. 1b showing the collapsed paraglacial deposits in the post-event image. The scar is ~36,000 m² in area and could have potentially contributed to the Dharali mudflow.

**Fig. 2:**
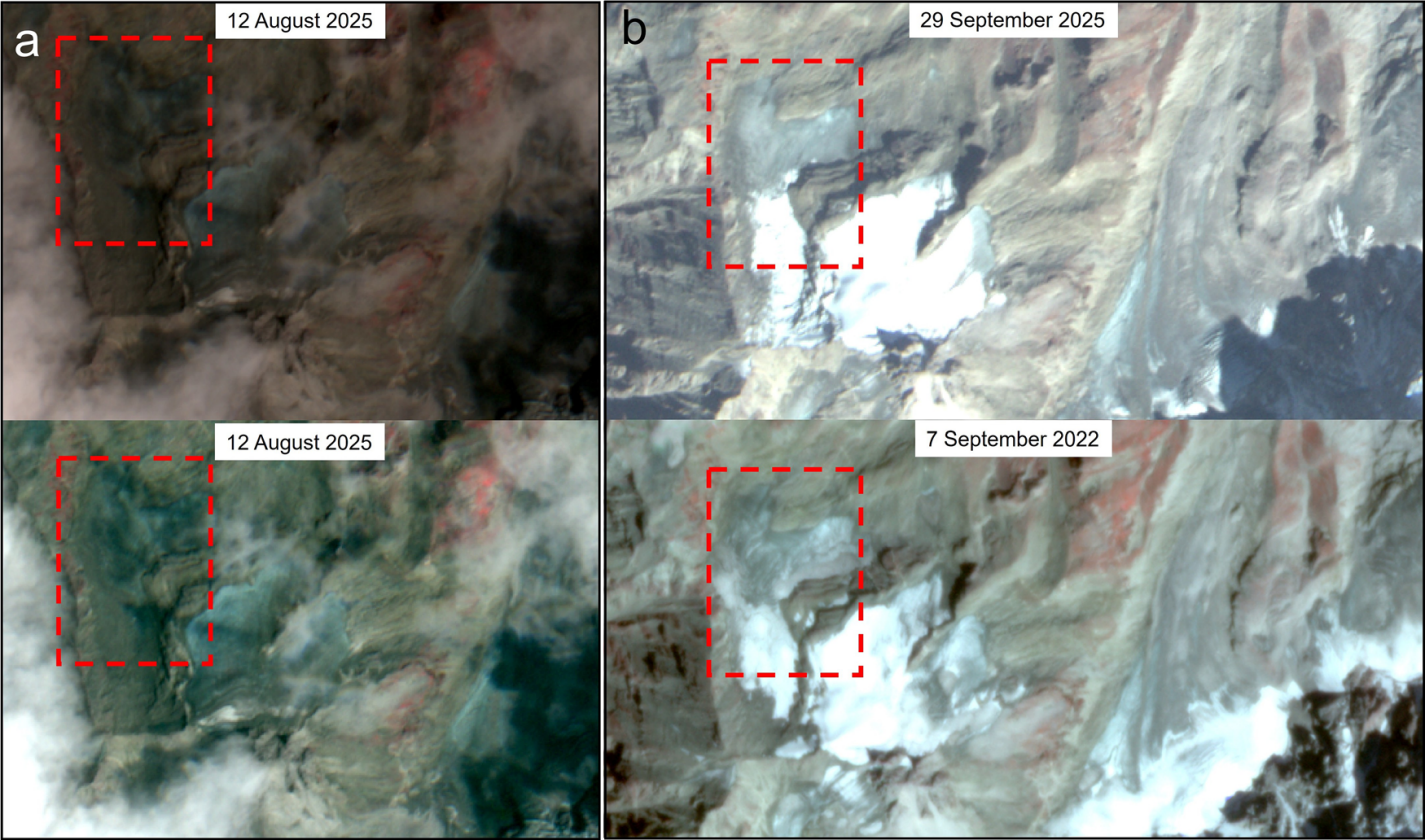
Visual comparisons. The red rectangle indicates the hanging glacier in question. **a** The 12 August 2025 image (LISS-IV; ID: R2F12AUG2025074282009600049SSANSTUC00GTDD; 5.8 m/pixel) shown before (top) and after (bottom) image enhancement. **b** Glacier visualization under the same illumination angle in two different years with nearly identical seasonal snow cover. The 5.8 m/pixel resolution 29 September 2025 image is from LISS-IV sensor (ID: R2F29SEP2025074964009600049SSANSTUC00GTDB) while the 3 m/pixel resolution 7 September 2022 image is from PlanetScope (ID: 2022-09-07_strip_5911352_composite).

Thus, three key points emerge from this analysis. (1) The ice body identified by Dandabathula et al.^1^ is not an ice patch but a typical hanging glacier. (2) If an ice patch had disappeared, as claimed by Dandabathula et al.^1^, it could not reappear within a month, as implied by the 22 September image shown in Fig. 1b, particularly during the summer season. Ice patches in mountain environments can regenerate or thicken only over at least one winter season, as they depend on the accumulation of winter snowfall to replenish mass lost during summer, followed by progressive compaction into ice. (3) In contrast, long-lived perennial ice bodies, such as the hanging glacier considered here, generally require hundreds to thousands of years to develop and reach a stable thickness. The enhanced image presented in Fig. 2b clearly shows the hanging glacier to be intact after the event and comparable in extent and morphology to the September 2022 image.

## Inadequate spectral evidence

The central claim of an “ice-patch collapse” by Dandabathula et al.^1^ rests primarily on post-event optical imagery from a single date; IRS ResourceSat-2 LISS-IV acquired on 12 August 2025. As evident in Fig. 4 of Dandabathula et al.^1^, this image is severely affected by cloud cover and poor illumination, resulting in suboptimal radiometric quality. The seasonal snow-free surface, combined with debris-mixed exposed ice, and dark patches caused by overhead cloud shadow, may create the impression that the ice has collapsed. Under such conditions, robust image preprocessing and enhancement, such as histogram stretching or matching, are essential for reliable surface interpretation. The glacier ice would have been identifiable had such standard radiometric corrections been applied. We used the same LISS-IV scene and performed appropriate image enhancement, which clearly reveals the glacier to be intact as the bluish tinge gets more prominent than the surroundings (Fig. 2a bottom panel).

To improve interpretability of the LISS-IV imagery, we applied a localized enhancement workflow focused on the glacier of interest. The image was first spatially subset to the area surrounding the ice body, and contrast was then enhanced using a standard deviation stretch (±2σ) applied to the bands within the ArcGIS 10.8.1 Symbology environment, which improves discrimination of ice and shadowed terrain under low illumination conditions. Additional contrast balancing was performed to minimize the influence of cloud shadow and terrain shading, allowing clearer identification of surface features such as ice margins. Moreover, the 29 September 2025 LISS-IV image in Fig. 2b further confirms that the glacier remained intact. Indeed, Fig. 2b demonstrates that glacier outlines from two images acquired in the same month, ensuring comparable illumination conditions and nearly identical seasonal snow cover, over a three-year interval show minimal to no change in glacier extent. Moreover, no quantitative spectral analysis is presented by Dandabathula et al.^1^ to confirm the presence of glacier ice. In high-mountain terrain, bright reflectance alone cannot distinguish clean ice from saturated sediment, fine-grained debris, or freshly exposed landslide material. Reliable discrimination requires validated indices such as the Normalized Difference Snow Index (NDSI) or shortwave infrared diagnostics^5,6^. No such analysis is shown.

## No volumetric or DEM-based confirmation of ice detachment

Attributing failure to ice collapse requires quantitative evidence of mass loss consistent with glacier or ice-patch detachment. Established studies of high-mountain ice–rock avalanches use digital elevation model (DEM) differencing to constrain source volume and confirm material removal^7^. No comparable DEM-based volumetric assessment is presented by Dandabathula et al.^1^. Without visible surface changes and elevation differencing, there is no evidence of a coherent ice mass detaching, nor any estimate of the ice thickness lost. The interpretation^1^, therefore, rests solely on post-event imagery rather than measurable mass wasting. While we acknowledge that for several weeks after the event, obtaining clear satellite images over the study region was not possible, some planned stereopair acquisition even in the summer of 2026 can confirm if actually any substantial ice collapse occurred. This also highlights why deriving hasty inferences after any disaster might not be a prompt approach, specially when there are significant data gaps. Numerical or empirical models might be helpful in further filling some of these critical gaps and making the inferences more robust.

## Geomorphic characteristics are inconsistent with large ice avalanches

**Fig. 3:**
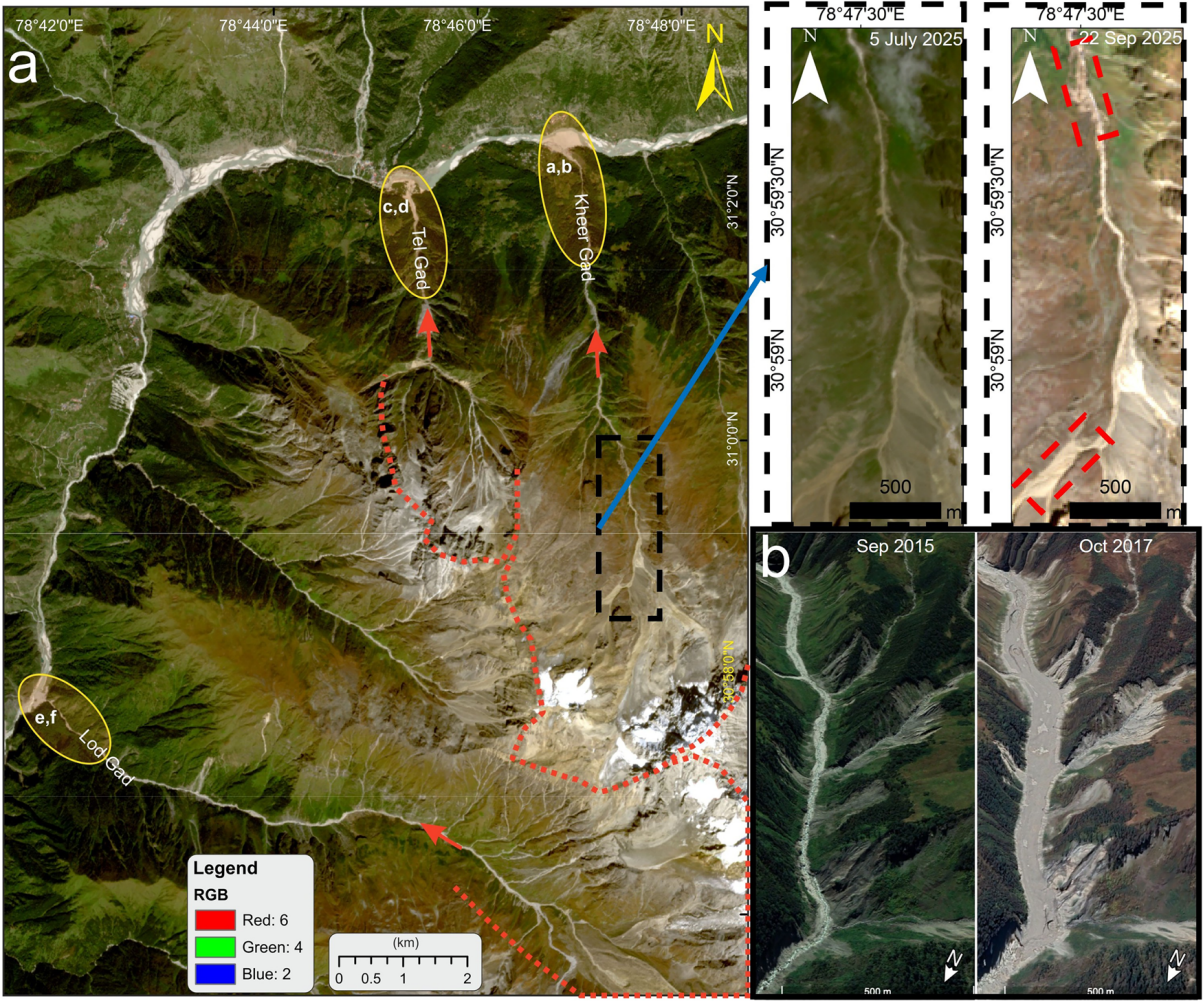
Channels showing erosion and deposition following recent mass movements. **a** Channels in the Bhagirathi region that experienced similar mudflows on 5 August 2025. The red dotted curves highlight the source regions of these three mudflows in the upper glaciated catchments. Red arrows indicate flow directions. Yellow ellipses mark the contextual locations of the field photographs of deposition areas shown in Fig. 4. The black rectangle shows the channel characteristics before and after the Dharali event. Red rectangles highlight regions displaying significant upslope and channel erosion. The 5 July 2025 image is 3 m/pixel resolution PlanetScope (Image ID: 20250705_055525_43_2538_3B_AnalyticMS_SR_8b) while the post-event image is 10 m/pixel resolution Sentinel 2 C (Image ID: S2C_MSIL2A_20250922T051701_N0511_R062_T44RKV). **b** An ice-rich avalanche in 2016 from the adjacent cliff that triggered the 2021 Chamoli event characteristically left spectrally identifiable ice remnants and blocky deposits along the runout in the Ronti Gad stream. These high-resolution images are taken from Google Earth (CNES/Airbus).

The reported Dharali source area lies within paraglacial terrain dominated by unconsolidated morainic and fluvio-glacial sediments^8^. The detachment morphology resembles slope failure within sedimentary material downslopes of the glacier rather than a fractured glacier margin. Moreover, ice-rich avalanches characteristically leave thermally and spectrally identifiable ice remnants or blocky deposits along the runout^9^. For example, an ice-rich avalanche in 2016 from the adjacent cliff that triggered the 2021 Chamoli event left typically discernible ice-debris deposits along the runout in the Ronti Gad stream (Fig. 3b), visible even after five years of the event^9^. No such ice-rich deposits could be observed in the post-event images of the Kheer Gad channel causing Dharali event (black rectangle in Fig. 3a). The depositional morphology described for Dharali is consistent with dense, sediment-dominated debris flow, not ice-dominated avalanche dynamics. Dense debris flows typically exhibit high-solid fractions and channelized levees formed through granular-fluid interactions^10^, rather than fragmented ice blocks. The geomorphic evidence, therefore, supports sediment failure rather than cryospheric detachment.

## Absence of supporting geophysical evidence

Large ice or rock–ice avalanches generate broadband seismic signals with characteristic frequency evolution and sustained duration^7,11^. No geophysical evidence is presented by Dandabathula et al.^1^ to corroborate a high-energy ice detachment. Cryospheric collapse of significant magnitude should produce a distinct seismic signature, particularly in the 1–10 Hz range typical of mass movements. In the absence of broadband, long-duration signals, process attribution to a major ice avalanche remains unsubstantiated.

## Hydrometeorological forcing provides a sufficient alternative explanation

The event occurred during active monsoon conditions. Persistent rainfall can rapidly elevate pore-water pressures in saturated paraglacial sediments, triggering landslides and high-solid-fraction debris flows without requiring glacial detachment^12,13^. Paraglacial slopes in the Himalaya are particularly susceptible to rainfall-induced destabilization due to steep channel gradients, substantial supply of unconsolidated glacial sediments from a retreating glacier, and evolving permafrost conditions. Under such conditions, rainfall-triggered failures represent a physically sufficient and well-documented mechanism.

**Fig. 4:**
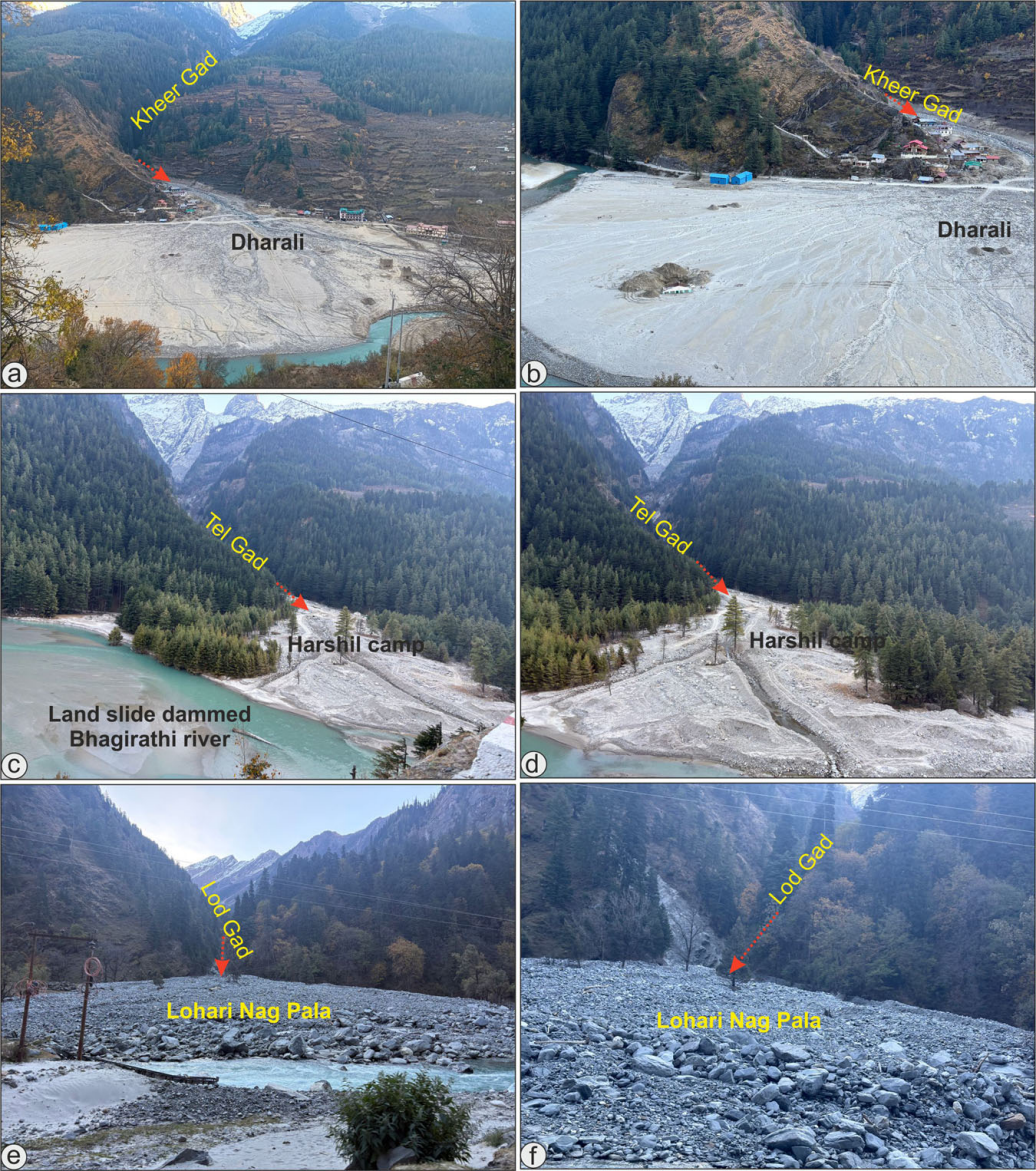
Field photographs of the deposition zones of the three channels with proximalsource zones which experienced near-synchronous mudflows on 5 August 2025. **a** An overview of the Dharali Village after the destructive mudflow. **b** Mudflow deposits at Dharali. **c** An overview of the Harshil Army Camp after the mudflow. **d** A closer look of the Tel Gad channel. **e** An overview of the Lohri Nag Pala after the debris flow. **f** Debris flow deposits at Lohari Nag Pala.

This mechanism provides a coherent explanation for the occurrence of multiple debris flows within ~5 h of the main Dharali (31° 2'26.05“N; 78°46'51.31“E) event in the Kheer Gad catchment, for the contemporaneous flash flood reported in the adjacent Tel Gad catchment, which impacted the Harshil Army Camp (31° 2'4.31“N; 78°45'17.10“E) located ~4 km downstream of Dharali, and for another one near Lohari Nag Pala (30°57'46.26“N; 78°41'52.01“E), downstream of Sukhi (Fig. 4). The near-synchronous nature and close spatial proximity of the source zones for these events are more consistent with a common hydrometeorological trigger upstream than with a localized, single-source cryospheric failure causing Dharali event^1^.

A rigorous process attribution framework requires systematic investigation of competing hypotheses for high-mountain mass movement events. For example, such debris flows can be caused by several triggers such as ice collapse, glacial lake outburst flood (GLOF), rock–ice avalanche, rainfall-triggered landslide, followed by progressive channel bulking. The published interpretation^1^ does not demonstrate such hypothesis testing, instead, it privileges a cryospheric explanation without excluding simpler rainfall-driven mechanisms. A recent article^8^ on this event is a good example of multiple possibility consideration, where the author has investigated rainfall triggered reactivation of older landslide, GLOF, and wind convergence, as several potential or interacting triggers.

Another analysis^14^ of the Dharali event and several similar events across the Uttarakhand state have proved that the frequency, magnitude, and direct loss due to landsliding in 2025 was significantly higher mainly due to intense rainfall and cloudbursts. The study^14^ reported that the variance of the rainfall data during August-September 2025 was relatively higher indicating intense and distinct rainfall peaks. The rainfall during June-September 2025 was also considerably higher throughout the state compared to previous years, including a total rainfall of 4808.6 mm in Gaurikund (Rudraprayag), 1321.6 mm in Kalagarh (Pauri Garhwal), 1547.8 mm in Pipalkoti (Chamoli), and 1701.8 mm in Uttarkashi^14^. All these places also experienced multiple landslides and mudflow events, thus, highlighting a potential atmospheric hydrometeorological trigger which was a large-scale phenomenon impacting several valleys across the state of Uttarakhand.

## Implications of incorrect process attribution

Mischaracterising a rainfall-triggered debris flow as an ice-collapse event has substantial implications for hazard assessment. Cryospheric triggers emphasize glacier instability monitoring, whereas rainfall-triggered failures highlight hydrometeorological thresholds, land-use regulation, and paraglacial sediment management. In rapidly urbanizing Himalayan valleys, incorrect attribution risks misdirecting early warning strategies, infrastructure design, and policy responses^15^. For example, focusing on glacier collapse may prioritise remote cryospheric surveillance while overlooking localized, short-duration intense rainfall events that are far more difficult to detect but critical for early warning.

From a scientific perspective, such misinterpretation can skew future research priorities, leading to overemphasis on relatively rarer or unsupported mechanisms while underinvesting in the study of rainfall–sediment interactions, hillslope saturation dynamics, and channelized debris flow processes. It may also propagate inaccuracies into regional hazard models, susceptibility maps, and risk forecasts, particularly where data are sparse and single-event interpretations disproportionately influence broader understanding.

The policy implications are equally significant. Hazard zoning, infrastructure planning, and disaster preparedness strategies depend on accurate identification of dominant processes. Misattribution may result in ineffective mitigation measures, such as neglecting no-build zones on historical debris flow fans or failing to implement rainfall-based early warning systems. In the context of expanding tourism and infrastructure in the Himalaya, this can translate directly into increased exposure and vulnerability of communities.

At a societal level, incorrect process attribution can distort risk perception among local populations and decision-makers, potentially fostering a false sense of security or misinformed preparedness strategies. Robust hazard science therefore requires integrating spectral validation, geomorphic analysis, hydrometeorological context, and geophysical constraints before concluding on the hazard process chain. Accurate process attribution is essential not only for understanding past events but also for building resilient, evidence-based responses to future mountain hazards.

## Data Availability

No datasets were generated or analysed during the current study.
